# The C-terminal region of the *Plasmodium berghei* gamete surface 184-kDa protein Pb184 contributes to fertilization and male gamete binding to the residual body

**DOI:** 10.1186/s13071-024-06374-7

**Published:** 2024-07-13

**Authors:** Kazuhiko Nakayama, Asako Haraguchi, Jun Hakozaki, Sakure Nakamura, Kodai Kusakisako, Hiromi Ikadai

**Affiliations:** Laboratory of Veterinary Parasitology, School of Veterinary Medicine, Kitasato University, Aomori, Towada 034-8628 Japan

**Keywords:** *Plasmodium berghei*, Gamete, Fertilization, Transmission block

## Abstract

**Background:**

Malaria, a global health concern, is caused by parasites of the *Plasmodium* genus, which undergo gametogenesis in the midgut of mosquitoes after ingestion of an infected blood meal. The resulting male and female gametes fuse to form a zygote, which differentiates into a motile ookinete. After traversing the midgut epithelium, the ookinete differentiates into an oocyst on the basal side of the epithelium.

**Methods:**

Membrane proteins with increased gene expression levels from the gamete to oocyst stages in *P. berghei* were investigated utilizing PlasmoDB, the functional genomic database for* Plasmodium* spp. Based on this analysis, we selected the 184-kDa membrane protein, Pb184, for further study. The expression of Pb184 was further confirmed through immunofluorescence staining, following which we examined whether Pb184 is involved in fertilization using antibodies targeting the C-terminal region of Pb184 and biotin-labeled C-terminal region peptides of Pb184.

**Results:**

Pb184 is expressed on the surface of male and female gametes. The antibody inhibited zygote and ookinete formation in vitro. When mosquitoes were fed on parasite-infected blood containing the antibody, oocyst formation decreased on the second day after feeding. Synthesized biotin-labeled peptides matching the C-terminal region of Pb184 bound to the female gamete and the residual body of male gametes, and inhibited differentiation into ookinetes in the in vitro culture system.

**Conclusions:**

These results may be useful for the further studying the fertilization mechanism of *Plasmodium* protozoa. There is also the potential for their application as future tools to prevent malaria transmission.

**Graphical Abstract:**

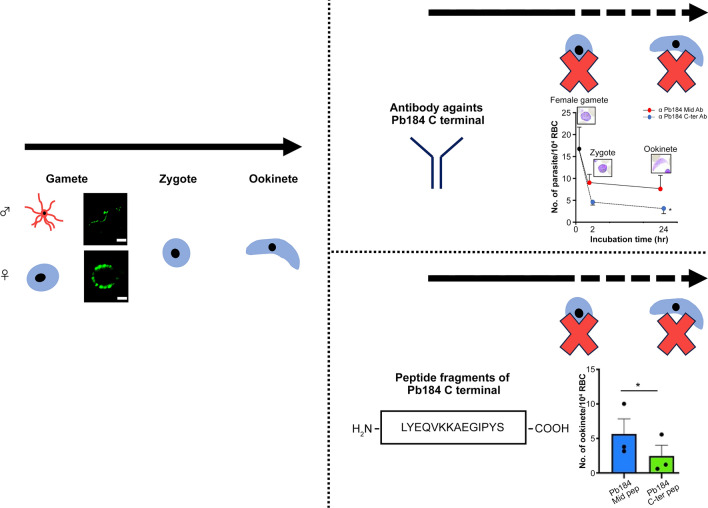

**Supplementary Information:**

The online version contains supplementary material available at 10.1186/s13071-024-06374-7.

## Background

Malaria is among the deadliest infectious diseases in the world. In 2022, over 200 million people were reportedly infected with malaria, leading to approximately 600,000 deaths [1]. Although the fight against the transmission of malaria has significantly progressed, the emergence and spread of drug-resistant parasites and insecticide-resistant mosquitoes has slowed down progress since 2015. To defeat malaria, it is essential to have a better understanding of parasite transmission mechanisms and then to develop new tools.

Malaria parasites belong to the genus *Plasmodium*, and their life-cycle can be divided into two stages: asexual reproduction within the human body and sexual reproduction within the mosquito body [2]. Male and female gametocytes form in human blood acquired by female *Anopheles* mosquitoes during a blood meal. In the mosquito midgut, the parasites undergo gametogenesis, forming male and female gametes. Male gametes detach from the exflagellation center and move into the mosquito blood bolus in search of female gametes. In the aftermath of the male gamete departure, the persisting structures are denoted as residual bodies [3]. Male and female gametes fuse to undergo fertilization, forming zygotes, which differentiate first into motile ookinetes, then into oocysts beneath the basal lamina upon traversing the midgut epithelium. Parasite numbers decrease by approximately thousandfold from gametes to oocyst formation [4]. Subsequently, thousands of sporozoites are formed inside each oocyst, expanding parasite numbers by several orders of magnitude [5].

Fertilization can be divided into two stages: male and female gamete adhesion, followed by membrane fusion. In the case of *Plasmodium berghei*, a rodent malaria parasite, several male and female gamete adhesion-related membrane proteins have been identified. These proteins, including Pbs48/45, Pbs230, Pb22 and Pb115, are expressed in male gametes [6–11]. Another protein, Pbs47, is expressed in female gametes [9], and the membrane protein PbHAP2/GCS1 has been identified during membrane fusion [12–14]. Antibodies against these proteins inhibit fertilization and reduce oocyst formation [13]. Notably, *Plasmodium falciparum* proteins Pfs48/45 and PfHAP2/GCS1 are orthologs to Pbs48/45 and PbHAP2/GCS1, respectively. Pfs48/45- and PfHAP2/GCS1-knockout *P*. *falciparum* parasites have been reported to exhibit functions similar to those of *P. berghei*. [8, 14], suggesting the presence of male and female gamete conjugation-involved membrane proteins [15]. Recently, female gamete-binding peptide (FG1), selected by phage display screening of *P.* *berghei* male gametes, was found to conform to heat shock protein 90 (HSP90) on the surface of female gametes and to competitively inhibit fertilization [16]. However, the molecular interacting (receptor–ligand) protein partners on the gamete surfaces remain unknown.

In this study, we searched the PlasmoDB (https://plasmodb.org/plasmo/app/) database, a functional genomic database for* Plasmodium* spp., for malaria parasite membrane proteins with increasing messenger RNA (mRNA) expression at ookinetes, with the aim to investigate unknown mosquito transmission-involved functional genes (80 genes). Of the products of the chosen 80 genes, we selected five Pb184-containing candidate proteins based on their high expression levels in oocysts. Among the encoding genes of the five selected proteins, we included those previously reported on the ookinete surface and oocyst capsule protein (OSCP) [17]. We identified a gene encoding a 184-kDa protein, Pb184 (PBANKA_1244800), generated antibodies targeting the Pb184 C-terminal region and then investigated the role of this protein in male and female gamete adhesion. Using C-terminal region-derived peptides, we demonstrated the binding of these peptides to the surface of both male and female gametes, thereby implying Pb184 involvement in gamete adhesion.

## Methods

### Parasites, mice and mosquitoes

Male BALB/c mice (SLC Inc., Hamamatsu, Shizuoka, Japan) aged 6–8-weeks were infected with wild-type (WT) *P. berghei* or with the constitutively green fluorescent protein (GFP)-expressing *P. berghei* ANKA strain [18]. *Anopheles stephensi* (STE2 strain) mosquitoes were maintained in an insectary at 27 °C and 80% relative humidity under a 14/10-h light/dark cycle and fed with a 10% (w/v) sucrose solution. For the mosquito infection experiments, we used *P. berghei*-infected mice with at least 10 exflagellations per 10^4^ red blood cells (RBCs).

### Sequence analysis

To identify potential gamete and ookinete surface protein-encoding genes, we searched the PlasmoDB malaria database for proteins expressed in gametocytes and ookinetes with at least one transmembrane domain. Our search yielded PBANKA_1244800, encoding Pb184, and a 184-kDa putative protein. We obtained orthologous sequences in *Plasmodium yoelii* (PY17X_1248300), *P. chabaudi* (PCHAS_1245200), *P. vinckei* (PVVCY_1204580), *P. falciparum* (PF3D7_0530400), *P. vivax* (PVP01_1003500), *P. ovale* (PocGH01_10011300), *P. malariae* (PmUG01_10014200), *P. knowlesi* (PKNK_1002300), *P. gaboni* (PGSY75_0530400) and *P. reichenowi* (PRCDC_0529500) upon further screening in PlasmoDB. We performed BLAST analysis on the PlasmoDB platform to determine amino acid (aa) sequence similarities.

### Anti-Pb184 antibody production and purification

**Fig. 1:**
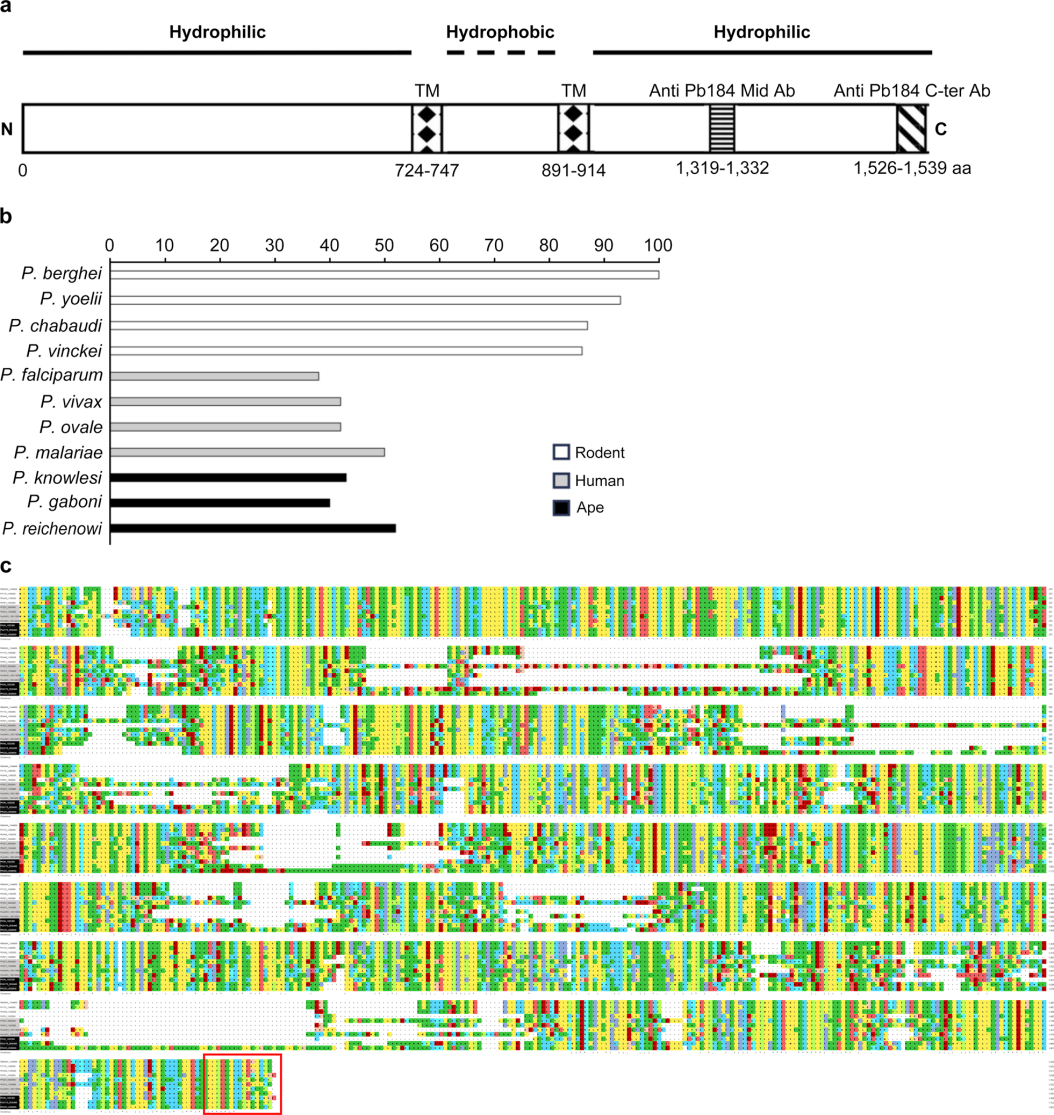
Analysis of the Pb184 protein sequence. **a** The Pb184 protein, predicted to be 1539 aa in length, contains 2 transmembrane domains within the regions 724–747 aa and 891–914 aa. Polyclonal Abs were raised against peptides from the 1329–1346 and 1526–1539 aa regions, designated as anti-Pb184 Mid Ab and anti-Pb184 C-ter Ab, respectively. **b** Percentage of aa sequence identity between Pb184 and orthologous sequences from other* Plasmodium* species. **c** Alignment of Pb184 aa sequences with those of other* Plasmodium* orthologs, highlighting the C-terminal region within a red box. aa, Amino acid; Ab, antibody; C, N, C and N-terminal regions, respectively; TM, transmembrane

Rabbit polyclonal antibodies were raised against the Pb184 protein middle (Pb184 Mid) and C-terminal (Pb184 C-ter) regions, corresponding to aa 1319–1332 and 1526–1539, respectively), using the epitope selection service provided by Eurofins Genomics Inc. (Tokyo, Japan) (Fig. 1a). We chose the selected regions based on their high-level antigenicity within the protein. The antibodies were purified using a saturated ammonium sulfate solution and the Ab-Rapid SPiN EX column (ProteNova, Tokyo, Japan), according to the manufacturer’s instructions, until equilibration. We designated the purified antibodies as Anti-Pb184 Mid and Anti-Pb184 C-ter antibodies (Abs).

### Biotin-labeled Pb184 peptide preparation

N-terminally biotinylated peptides were synthesized from the middle or C-terminal region (Biologica Inc., Nagoya, Japan), and denoted as negative and C-terminal peptides (biotin-labeled Pb184 Mid-Pep: 1319–1332 aa; biotin-labeled Pb184 C-ter-Pep: 1526–1539 aa), respectively. The peptides were dissolved in phosphate-buffered saline (PBS) solution at a concentration of 10 μg/μl for further use.

### Parasite preparation

#### Schizont

The schizont culture and purification methods were performed as described previously [19]. Briefly, we obtained 1–3%-parasitemia-infected blood from mice through a cardiac puncture. The collected blood was subsequently introduced into the schizont culture medium, consisting of RPMI 1640 medium supplemented with 10% (vol/vol) heat-inactivated fetal bovine serum (FBS), and incubated overnight at 37 °C. Following incubation, the culture was fractionated using a 55% Nycodenz pad to isolate schizont-stage parasites (final Nycodenz concentration: 15%).

#### Gametocytes

Gametocytes were purified as described previously [20]. Briefly, we inoculated 10^6^ *P. berghei*-infected erythrocytes into mice and injected pyrimethamine (0.5 mg/ml) in PBS/0.05% Tween 20 intraperitoneally in the mice on days 4 and 5 post-infection. We verified gametocytemia using Giemsa-stained tail blood smears 24 and 48 h after the pyrimethamine treatment.

### Exflagellation analysis

Male gametocyte exflagellation was verified as previously described [9]. Briefly, we collected 2 μl of infected blood from the tail vein and immediately mixed it with 38 μl of complete ookinete culture medium (OKM: RPMI 1640 medium [Gibco, Thermo Fisher Scientific, Waltham, MA, USA] with 20% heat-inactivated FBS [Sigma-Aldrich, St. Louis, MO, USA]). We placed the mix under a coverslip at 19 °C for 15 min and counted exflagellation centers/10^4^ erythrocytes using a Thomas hemocytometer (Nippon Rinsho Kikai Kogyo Co., Ltd., Tokyo, Japan).

### Ookinete culture and purification

Blood was obtained from mice with exflagellation of 10/10^4^ erythrocytes through cardiac puncture. We supplemented the blood samples with 10 volumes of OKM and cultured the blood for 24 h at 19 °C. The ookinetes were purified using a MidiMACS separator system (Miltenyi Biotec, Bergisch Gladbach, Germany) as described previously [21]. A total volume of 3 ml of culture was passed three times through the column before column removal from the magnet. We recovered the ookinetes by passing 5 ml of OKM through the column, then centrifuging the purified ookinetes at 1000 *g* for 10 min at 4 °C, followed by washing 3 times with PBS.

### Quantitative reverse transcription PCR

To prepare the samples, first, we performed leukocyte removal and hemolysis. Leukocyte removal was as described previously [22]. Briefly, we filled a 10-ml syringe with round-cut filter paper, placing the paper inside the syringe to cover the outlet lumen. Next, we added CF11 cellulose powder (Whatman®, Whatman plc, Maidstone, UK) to the syringe, packed it down to obtain 3 ml of densely packed cellulose and then wet the CF11 column with approximately 5 ml of PBS solution. We then added and collected gamete-induced blood in 6 ml of PBS and centrifuged the blood sample at 3000 *g* to obtain a pellet. The hemolysis treatment was performed continuously by adding saponin to the leukocyte-free blood pellet. We added 0.15% saponin/PBS to increase the volume of the pellet by threefold, followed by resuspension; we then allowed the mixture to stand on ice for 10 min, centrifuged the sample at 3000 *g*, removed the supernatant and supplemented it with the saponin solution again, followed by the same treatment. Finally, we washed the sample three times with 1× PBS, then removed the supernatant and stored it at − 80 °C until further use.

We extracted total RNA from the schizont, gamete and ookinete samples using NucleoSpin RNA Blood Kit (Takara Bio, Otsu, Japan). Reverse transcription-PCR (RT-PCR) was performed using the ReverTra Ace qPCR RT Master Mix with gDNA Remover (Takara Bio) and the real-time RT-PCR reactions were performed using the KOD SYBR® qPCR Mix (Toyobo, Osaka, Japan) according to the manufacturer’s instructions. The sequences of the primers we used in this study are summarized in Additional file 1: Primer list. Each reaction was performed in a total reaction mix of 10 µl (1 µl cDNA, 0.2 µl each of the 10 mM forward and reverse gene-specific primers, 3.4 µl water, 0.2 µL ROX, and 5 µl SYBR-green) using the StepOnePlus™ Real-Time PCR System (Applied Biosystems, Thermo Fisher Scientific). We normalized the relative gene expression levels to that of the 18S rRNA (PBANKA_1237000) and compared them using the standard curve method.

### Western blotting

Protein was extracted from gamete-induced samples by supplementing the pellet with RIPA buffer (50 mM Tris–HCl, 150 mM NaCl, 1% NP-40, 0.5% sodium deoxycholate, 0.1% SDS and 1 mM EDTA, pH 7.4) and rotating at 4 °C for 30 min. Protein concentration was measured using the Bradford method and 20 µg protein lysates were separated in 6% or 12% sodium dodecyl sulfate polyacrylamide gel electrophoresis (SDS-PAGE) gels. For western blotting, we first transferred the protein samples onto a 0.45-µm polyvinylidene difluoride membrane (Bio-Rad Laboratories, Hercules, CA, USA), then we blocked the membrane using 3% skim milk in PBS buffer for 1 h, followed by probing it with anti-Pb184 C-ter serum or anti-Pb184 Mid serum at 1:500 for 2 h. We used mouse anti-PbLDH Ab (1:1000) as a control to estimate protein loading. The anti-PbLDH Ab was raised by NITTOBO MEDICAL Co., LTD (Tokyo, Japan). After washing 3 times with PBS buffer containing 0.1% Tween 20 (PBST), we labeled the bound primary antibodies with horseradish peroxide (HRP)-conjugated goat anti-rabbit or -mouse antibodies (Invitrogen, Thermo Fisher Scientific) diluted 1:2000 in PBST. After washing the blots 3 times with PBST, we visualized the proteins on the blot using ImmunoStar Zeta (Fuji Film, Tokyo, Japan).

### Fluorescence assays

The purified gametes and cultured ookinetes were placed on MAS-coated glass slides (Matsunami Glass, Yamagata, Japan) for 30 min at 4 °C, fixed with 4% paraformaldehyde (PFA)/PBS at 24 °C for 10 min, then washed 3 times with PBS for 5 min each time. The gamete samples were prepared by first incubating the samples in OKM at 19 °C for 15 min, followed by incubation with 0.1 M glycine (Fujifilm Wako Pure Chemical, Tokyo, Japan) for 10 min and then three washes with PBS for 5 min each wash. To verify protein localization on the membrane, we either permeabilized the samples with 0.1% Triton X-100/PBS for 10 min or processed them directly without permeabilization. Next, we blocked the samples with 2% bovine serum albumin (BSA)/PBS for 1 h at 24 °C. For the immunofluorescence assay (IFA), we incubated the primary antibody (i.e. anti-Pb184 serum diluted 750-fold with 1% BSA/PBS) overnight at 4 °C, then washed it 3 times with 0.05% PBST for 5 min each time. We used Alexa Fluor® 488 goat antirabbit immunoglobulin (IgG; H + L) (Thermo Fisher Scientific) as a secondary antibody, diluted 1000-fold in 1% BSA/PBS, allowing it to react for 1 h at 24 °C; nonimmunized serum was used as a negative control. After washing with PBST, we implemented an additional membrane permeabilization process using a solution of PBS containing 0.05% Triton X100 in the case of samples that had not been subjected to membrane permeabilization. We added anti-α-tubulin mouse Ab and anti-OSCP rabbit serum [17] as stage-specific markers for male gametes and oocysts, respectively. Next, we incubated the samples with the secondary antibody, Alexa Fluor® 568 goat antirabbit IgG (H + L) (Thermo Fisher Scientific) diluted 1000-fold in 1% BSA/PBS, for 1 h at 24 °C, followed by three washes with PBST for 5 min each time. The parasites were mounted in lowFade® Diamond Antifade Mountant with DAPI (Molecular Probes, Eugene, OR, USA) and observed them using an Eclipse E600 (Nikon, Tokyo, Japan) fluorescence microscope. For fluorescence staining with peptide proteins, we reacted 0.2 μg/μl biotin-labeled Pb184 C-ter-Pep. instead of the primary antibody at 4 °C overnight. After washing 2 times with PBST, we added 100-fold-diluted Alexa568-labeled streptavidin (Thermo Fisher Scientific) to the samples and allowed them to react for 1 h at 24 °C.

### Enzyme-linked immunosorbent assay

The protein samples were extracted from the cytosolic and plasma membrane fractions separately. The cytoplasmic fraction was prepared by repeating 3 freeze–thaw cycles using a low-tension buffer (10 mM HEPES and 10 mM KCl, pH 7.4), centrifugation at 15,000 *g* for 30 min at 4 °C and collection of the supernatant. We prepared the membrane fractions by adding RIPA buffer (50 mM Tris–HCl, 150 mM NaCl, 1% NP-40, 0.5% sodium deoxycholate, 0.1% SDS and 1 mM EDTA, pH 7.4) to the remaining pellet and rotating at 4 °C for 30 min. We coated flat-bottomed 96-well enzyme-linked immunosorbent assay (ELISA) plates (Greiner Bio-One, Kremsmünster, Austria) in triplicate (6 μg/ml) with schizont or gamete protein samples in a coating buffer (15 mM Na_2_CO_3_ and 35 mM NaHCO_3_, pH 9.6) overnight at 4 °C. The plates were then washed 3 times with 0.05% PBST, blocked with 1% BSA for 1 h at 24 °C and incubated with biotin-labeled Pb184 Mid-Pep or biotin-labeled Pb184 C-ter-Pep for 16 h at 4 °C. The plates were then washed 3 times with PBST, incubated with 1 μg/ml HRP-conjugated streptavidin (Proteintech, Rosemont, IL, USA) for 1 h at 24 °C and washed again; detection was performed using 100 µl of o-phenylenediamine dihydrochloride/well (Sigma-Aldrich; 1 tablet/10 ml substrate solution containing 5150 μl of 0.1 M Na_2_-phosphate solution, 4850 µl of 0.1 M citric acid solution and 10 µl of hydrogen peroxide). After incubation for 30 min, absorbance was read at 450 nm using a VersaMax microplate reader (Emerson, St. Louis, MO, USA).

### Zygote-ookinete differentiation assay

The experimental design included two main groups: an anti-Pb184 Mid-Ab mixed control and an anti-Pb184 C-ter Ab mixed group. Each group was further subdivided based on time points, i.e. 0, 2, and 24 h (corresponding to female gametocytes, zygotes and ookinetes, respectively), resulting in three subgroups per group for technical replications. We selected parasitemia levels of approximately 10% and exflagellation rates of > 10/10^4^ for this experiment. At the 0 h time point, we prepared three blood smears and fixed them with methanol. Each Eppendorf tube was filled with 45 μl of OKM and mixed thoroughly with 10 μg of antibody. Subsequently, we added 5 μl of blood to each tube, incubated the tube for 2 h, collected the samples, then subjected the samples to a brief centrifugation step and discarded 45 μl of the supernatant. The smears were pipetted onto glass slides and fixed with methanol. Similar to the procedure just reported, we collected the samples at the 24 h time point, fixed them with methanol and subjected them to Giemsa staining for 15 min. The differentiation assay consisted of adding 10 μg of peptide protein at 0 h and comparing ookinete numbers at the 24 h time point.

### Ookinete gliding speed

Ookinete movements were studied by mixing ookinetes with Matrigel (Corning Inc., Corning, NY, USA), then measuring their displacement velocity [22–24]. We mixed purified ookinete suspensions containing 25-fold diluted serum and Matrigel in equal amounts, then applied 15-µl drops of the suspension to glass slides, covered the drops with glass coverslips (18 × 24 mm) and allowed the samples to stand for 20 min at 19 °C. Ookinete movements were then observed for 2 min using a digital microscope (KH-8700; Hirox, Tokyo, Japan) and the movement trajectories imaged for 10 min.

### Transmission-blocking experiment using antibodies

Mice that showed exflagellation of at least 10/10^4^ RBCs 5 days post-infection were used in these experiments [22]. A total of 50 female mosquitoes (pre-group) were allowed to feed on the infected mice for 15 min. The mice were then injected intravenously with 200 µg of anti-Pb184 C-ter Ab. After 5 min, another 50 mosquitoes (post-group) were allowed to feed on the same mice for 15 min. We then counted the number of oocysts at 2 or 14 days later. For the TB experiment 2 days after the blood meal, we repeated the oocyst counts after washing the guts with PBS for 1 min to remove any attached ookinetes.

### Statistical analyses

Welch’s t-test was used to compare the groups (quantitative PCR, number of exflagellations and ookinete speed). Welch’s analysis of variance (ANOVA) was used to compare the ookinete speed. The ratio paired-samples t-test was used to compare the number of zygotes and ookinetes, and the Mann–Whitney U-test was used to compare oocyst/midgut numbers. All analyses, except for the *U*, *Z* and *P* values of the Mann–Whitney U-test, were performed using GraphPad Prism software (GraphPad Software, San Diego, CA, USA). The *U*, *Z* and *P* values of the Mann–Whitney U-test were calculated using R software ® Foundation for Statistical Computing, Vienna, Austria).

## Results

### P184 is highly conserved within the *Plasmodium* genome

PBANKA_1244800 (Pb184) was identified through our in silico analysis using the PlasmoDB platform. Pb184 possesses at least one predicted transmembrane domain, conserved within the *Plasmodium* genus, and it belongs to a gene group that shows increasing expression from the gametocyte to the oocyst stages compared with the asexual stages. Pb184 is located on chromosome 12 and encodes a 1539-aa and 184-kDa protein, displaying transmembrane (TM) regions at aa positions 724–747 and 891–914, which predicts that both the N- and C-termini are exposed to the extracellular space (Fig. 1a). We detected no previously identified domain sequences. The Pb184 aa sequence is relatively conserved among different parasite species, with > 80% identity between *P. yoelii*, *P. chabaudi* and *P. vinckei*, and > 35% identity in human- and nonhuman primate-infecting species (Fig. 1b, c; Additional File 2).

**Fig. 2:**
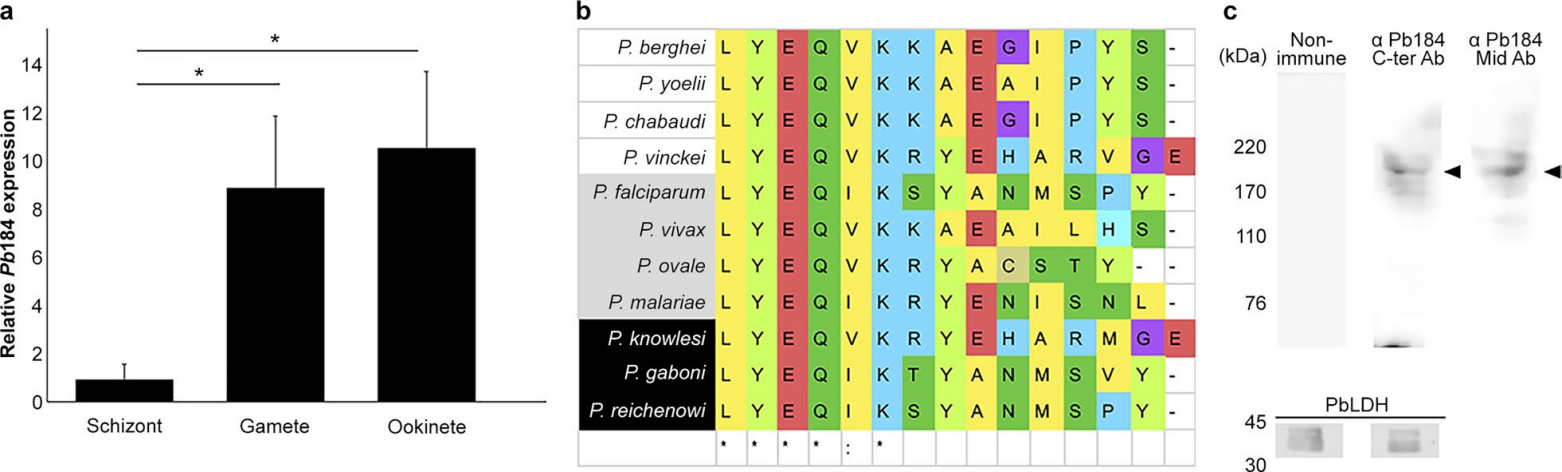
Expression and antibody production of Pb184. **a** Relative expression of Pb184 mRNA in schizonts, gametes and ookinetes, measured via real-time RT-PCR and normalized to 18S rRNA levels. Error bars represent standard errors calculated using one-way ANOVA with Tukey’s multiple comparisons test (*P < 0.05). **b** Amino acid sequence variations in the Pb184 C-ter region. Consensus areas are marked: “*” indicates identical residues, and “:” indicates similar residues.* Plasmodium* species infecting mice, humans and primates are depicted against white, gray, and black backgrounds, respectively. **c** Representative western blot of a gamete parasite lysate (20 μg protein), fractionated by gel electrophoresis and probed with anti-Pb184 C-ter Ab (1:500) or anti-Pb184 Mid Ab (1:500). The arrowheads indicate the band at around 184 kDa. Anti-PbLDH Ab (1:1,000) served as the loading control. Ab, Antibody; ANOVA, analysis of variance; mRNA, messenger RNA; Pb184 Mid, Pb184 C-ter, PB184 middle and C-terminal regions, respectively; rRNA, ribosomal RNA; RT-PCR, reverse-transcription PCR

Our *Pb184* gene expression-related quantitative-PCR experiments revealed *Pb184* upregulation in gametes and ookinetes compared with schizonts (Fig. 2a). We generated an anti-Pb184 C-ter Ab, targeting the conserved C-terminal region (Fig. 2b). The anti-Pb184 C-ter Ab detected an approximately 184-kDa protein in our western blots (Fig. 2c).

### Pb184 is present on the cell surface of both male and female gametes and ookinetes

**Fig. 3:**
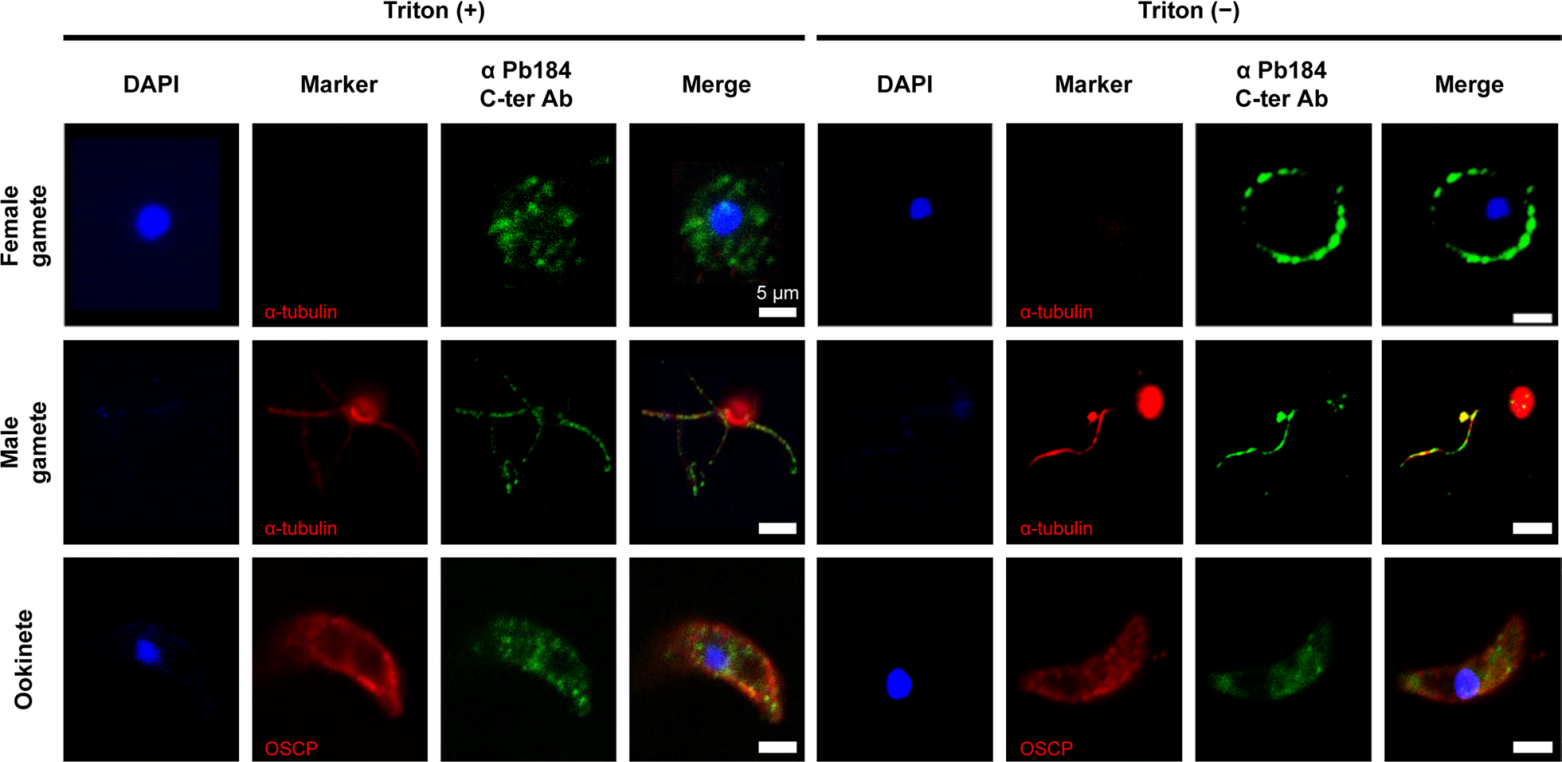
Localization of Pb184 protein by immunofluorescence assay. Representative photomicrographs depicting fluorescent staining of wild-type parasites at various developmental stages. Parasites were incubated with anti-Pb184 C-ter Ab (1:500) as the primary antibody (green), following a pre-treatment with (left panel) or without (right panel) Triton X-100. Additional staining included marker antibodies α-tubulin or OSCP (red), and nuclei were stained with DAPI (blue). All images were acquired under consistent conditions. Scale bar: 5 μm. DAPI, 4′,6-diamidino-2-phenylindole (fluorescent stain); OSCP, ookinete surface and oocyst capsule protein; Pb184 Mid, Pb184 C-ter, Pb184 middle and C-terminal regions, respectively;

Pb184 is a membrane protein with two predicted TM regions, and its mRNA abundance increases from asexual to sexual stages in mosquitoes. To confirm the potential Pb184 localization on the cell surface of gametes and ookinetes, we performed IFA using the anti-Pb184 C-ter Ab. Our results demonstrated that Pb184 was present on the cell surface of nonpermeabilized parasites (Fig. 3, right panel), exhibiting punctate localization both on male and female gametes and ookinetes. These results confirmed Pb184 localization on male and female gamete and ookinete cell membranes with an extracellularly exposed C-terminal region.

### Anti-Pb184 C-ter antibodies inhibit male and female gamete adhesion

**Fig. 4:**
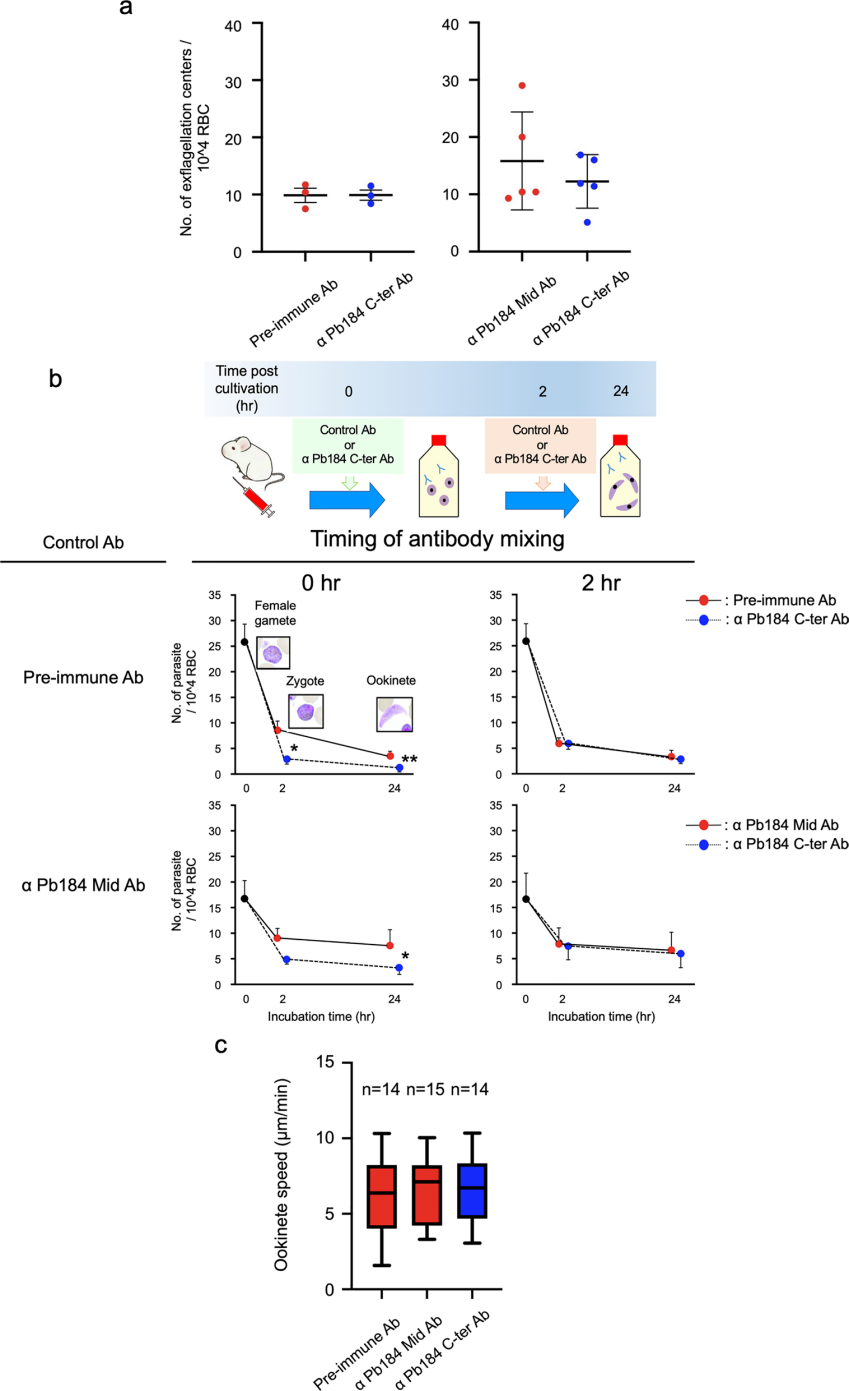
Phenotypic analyses using antibodies. **a** Number of exflagellation centers per field at 400× magnification. The left graph shows the results using pre-immune Ab as the control, with error bars representing the standard error (SE) from 3 biological replicates. Conversely, the right graph displays the results using anti-Pb184 Mid Ab as the control, with error bars indicating the SE across 5 biological replicates. **b** In vitro assay of zygote and ookinete differentiation, with antibodies added at the beginning (left panel) or after 2 h (right panel) of incubation. Zygote and ookinete formations were assessed after 2 and 24 h of incubation, respectively. The upper graphs present results using pre-immune Ab as the control, with error bars representing the SEs from 3 biological replicates each. The lower graphs show results using anti-Pb184 Mid Ab as the control, with error bars indicating the SEs from 4 and 3 biological replicates, respectively. Asterisks indicate a significant difference at *P < 0.05 and **P < 0.01 (ratio paired-samples t-test). **c** Ookinete gliding speed assessed in vitro. The left graph shows the results using pre-immune Ab as the control, while the right graph features result using anti-Pb184 Mid Ab as the control. Error bars represent the 10–90% percentile range from 3 biological replicates. Ab, Antibody; Pb184 Mid, PB184 Mid region; RBC red blood cell

Initially, we examined how the anti-Pb184 C-ter Ab could affect exflagellation and observed no significant effects compared with the negative control (Fig. 4a; pre-immune vs Pb184: Welch’s t-test, *t*_(3.64)_ = 0.022, *P* = 0.98; anti-Pb184 Mid vs Pb184: Welch’s t-test, *t*_(6.20)_ = 0.82, *P* = 0.44). Anti-Pb184 Mid Ab did not significantly reduce the number of ookinetes formed in the in vitro culture system compared to PBS and pre-immune antibodies; consequently, we used this antibody alongside pre-immune antibodies as a negative control (Additional File 3). Subsequently, we performed a fertilization-to-ookinete differentiation assay to investigate whether the anti-Pb184 C-ter Ab affects zygote and ookinete formation. We observed a slight and a significant reduction in zygote and ookinete formation, respectively, at the beginning of the incubation with anti-Pb184 C-ter Ab compared with that of the anti-Pb184 Mid Ab control (Fig. 4b; anti-Pb184 Mid Ab [lower graphs], ratio paired-samples t-test, *t*_(3)_ = 2.56 and 3.86, *P* = 0.082 and 0.036). Even when using pre-immune rabbit antibodies as a control, a significant reduction in the formation of zygotes and ookinetes was noted (Fig. 4b; pre-immune Ab [upper graphs], ratio paired-samples t-test, *t*_(2)_ = 7.44 and 19.3, *P* = 0.018 and 0.0027). Importantly, we detected no difference when the antibodies were mixed after 2 h of incubation (i.e. once fertilization had already occurred). Our ookinete motility assay revealed no significant impact on the motility speed of the anti-Pb184 C-ter Ab (Fig. 4c; Welch’ ANOVA, *F*_(2.00, 26.28)_ = 0.079, *P* = 0.92).

**Fig. 5:**
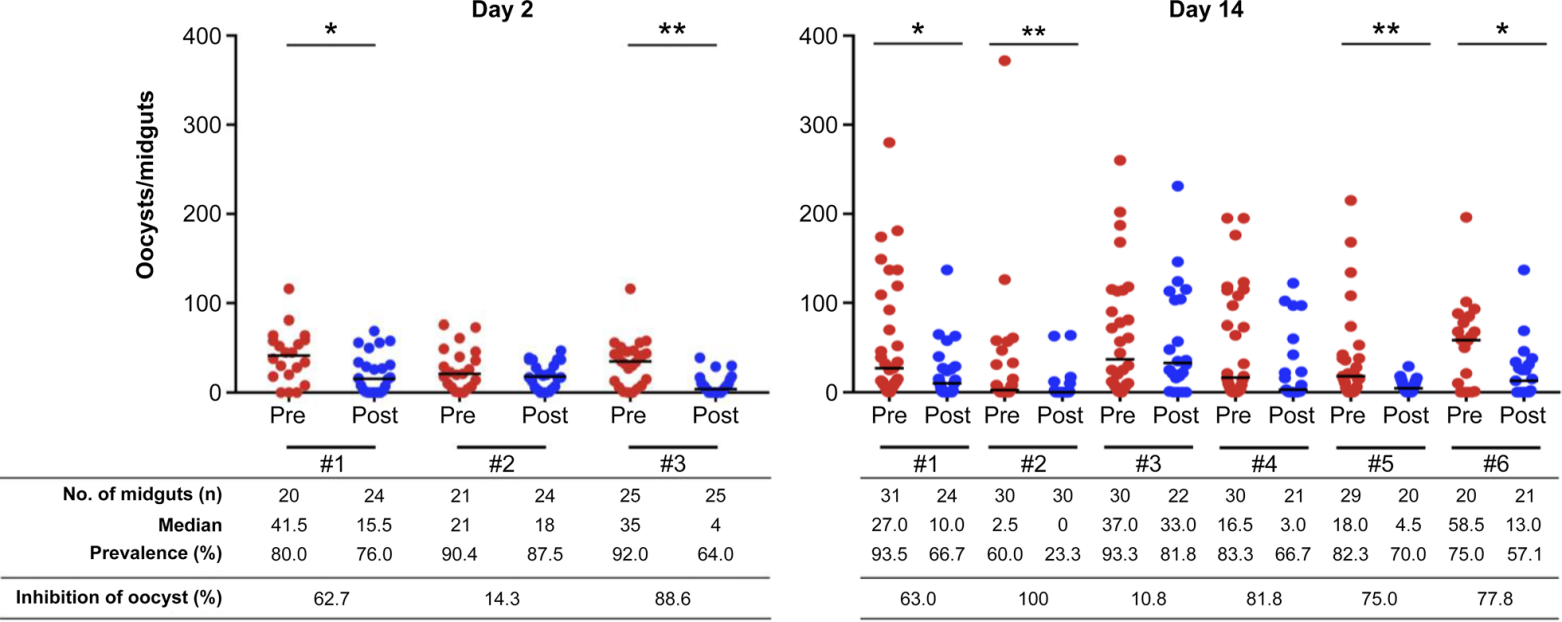
Transmission-blocking efficacy of anti-Pb184 C-ter Ab. Mosquitoes were allowed to feed on infected mice before (*Pre*) and after (*Post*) the intravenous injection of anti-Pb184 C-ter Ab. Oocysts were counted two or 14 days after feeding, as indicated. Asterisks indicate a significant difference at *P < 0.05 and **P < 0.01 (Mann–Whitney U-test). Ab, Antibody; Pb184 C-ter, Pb184 C-terminal region

As an alternative to investigating how the anti-Pb184 C-ter Ab affects parasite development in mosquitoes, we fed the mosquitoes prior to and after passive immunization of *P*. *berghei*-infected mice by the intravenous injection of anti-Pb184 C-ter Ab and then quantified oocyst formation. We discovered that the antibody significantly reduced oocyst formation on day 2 or on day 14 post-feeding (Fig. 5), indicating that the anti-Pb184 C-ter Ab suppressed parasite transmission to mosquitoes. The statistical analysis revealed the following results: Mann–Whitney U-test, Day 2: #1 *U*_(44)_ = 335.50, *Z* = 2.25, *P* = 0.025, #2 *U*_(45)_ = 292, *Z* = 0.90, *P* = 0.37, #3 *U*_(50)_ = 515.5, *Z* = 3.95, *P* < 0.001; Day 14: #1 *U*_(55)_ = 512.5,* Z* = 2.38, *P* = 0.017, #2 *U*_(60)_ = 609, *Z* = 2.59, *P* = 0.0096, #3 *U*_(52)_ = 345, *Z* = 0.27, *P* = 0. 79, #4 *U*_(51)_ = 398, *Z* = 1.59, *P* = 0.11, #5 *U*_(49)_ = 418, *Z* = 2.60, *P* = 0.009, #6 *U*_(41)_ = 288.5, *Z* = 2.07, *P* = 0.037. Taken together, the results of these experiments suggest that the Pb184 C-terminal region could be involved in fertilization.

### Pb184 C-ter region peptides bind to male and female gamete residual bodies

**Fig. 6:**
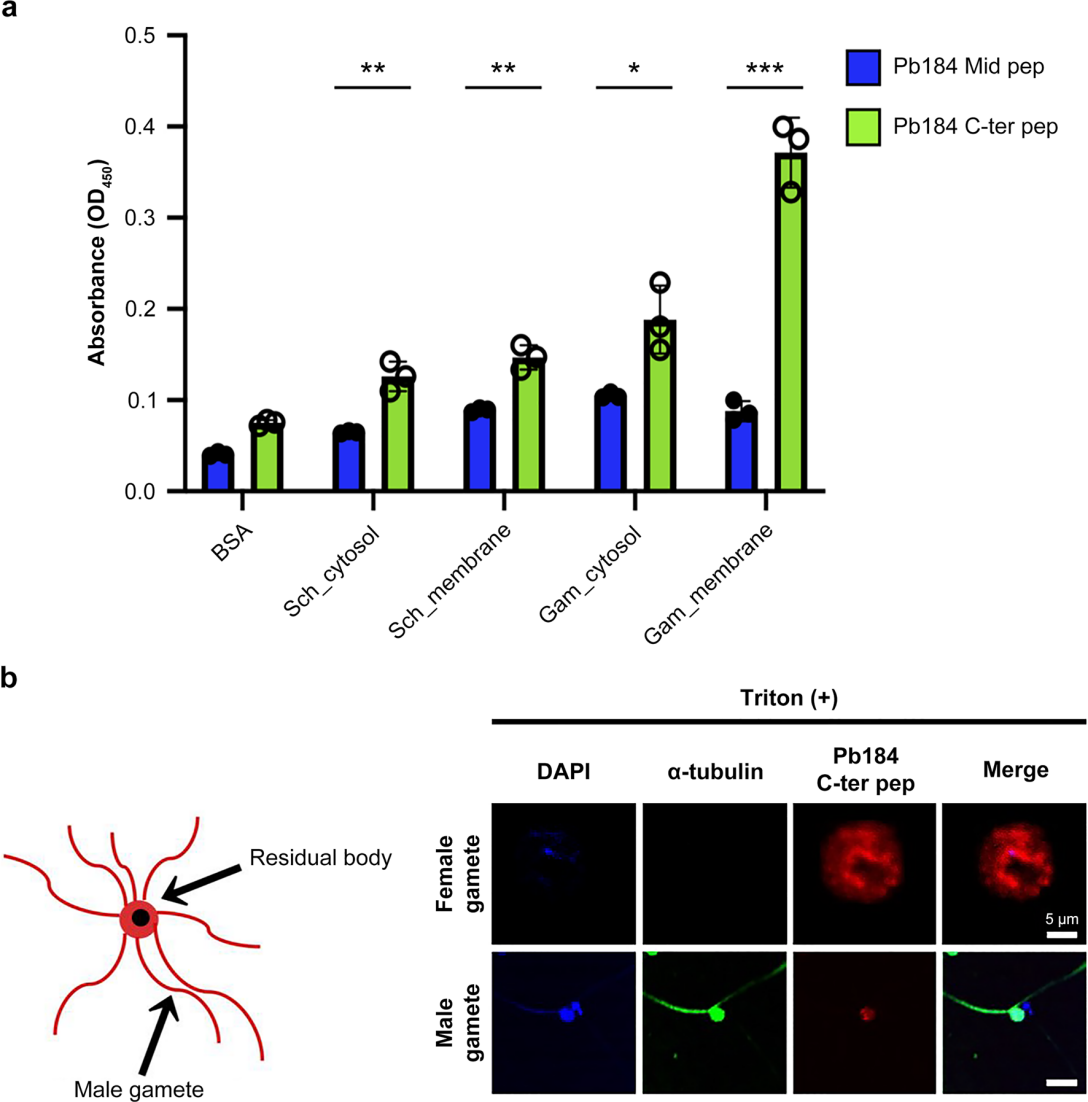
Gamete-binding affinity assessment using biotin-labeled Pb184 C-ter-Pep. **a** ELISA assessment of binding affinity. Plate wells were coated with BSA, schizont cytosol (*Sch_cytosol*), schizont membrane (*Sch_membrane*), gamete cytosol (*Gam_cytosol*) and gamete membrane (*Gam_membrane*), followed by reaction with biotin-labeled Pb184 Mid-Pep (blue) or biotin-labeled Pb184 C-ter-Pep (green). Asterisks indicate a significant difference at *P < 0.05, **P < 0.01 and ***P < 0.001 (Welch’s t-test) **b** Fluorescence staining assessment of binding affinity. Left: Illustration: male gametocyte residual body and male gamete. Male gametes persistently adhered to male gametocyte residual bodies 10–20 min following gamete induction. Right: Representative photomicrographs of male and female gamete fluorescent staining. Parasites were incubated with permeabilized (+ Triton X-100) and biotin-labeled Pb184 C-ter-Pep followed by Alexa 568 conjugated streptavidin (red). Anti-tubulin antibodies (green) were used as male gamete markers and nuclei were stained with DAPI (blue). All images were obtained under the same conditions. Scale bar: 5 μm. BSA, Bovine serum albumin; DAPI, 4′,6-diamidino-2-phenylindole (fluorescent stain); ELISA, enzyme-linked immunosorbent assay; Pb184 C-ter-Pep, Pb184 C-terminal peptide fragment; Pb184 Mid-Pep, Pb184 middle peptide fragment

The fact that the anti-Pb184 C-ter Ab recognizes both male and female gametes raises the possibility that Pb184 contributes to male–female gamete binding at fertilization. We thus produced biotin-labeled peptide fragments (biotin-labeled Pb184 C-ter-Pep) to investigate this hypothesis. Our ELISA results yielded strong binding between biotin-labeled Pb184 C-ter-Peps and gamete cell membrane fractions compared with the obtained results anti-Pb184 Mid-Ab recognition region peptide (biotin-labeled Pb184 Mid-Pep) (Fig. 6a). As shown in Fig. 4b, since anti-Pb184 Mid Ab did not affect the differentiation into ookinetes, we used Pb184 Mid Pep as a negative control. Subsequently, we conducted a localization analysis using fluorescence staining of the peptides. Our results revealed that biotin-labeled Pb184 C-ter-Pep binds both to female gametes (Fig. 6b) and male gamete residual bodies. These results suggest that the Pb184 C-terminal region could be potentially involved in binding to specific proteins in female gametes as well as in binding between male gametes and residual bodies.

### Pb184 C-terminal region peptide inhibits male–female gamete adhesion

**Fig. 7:**
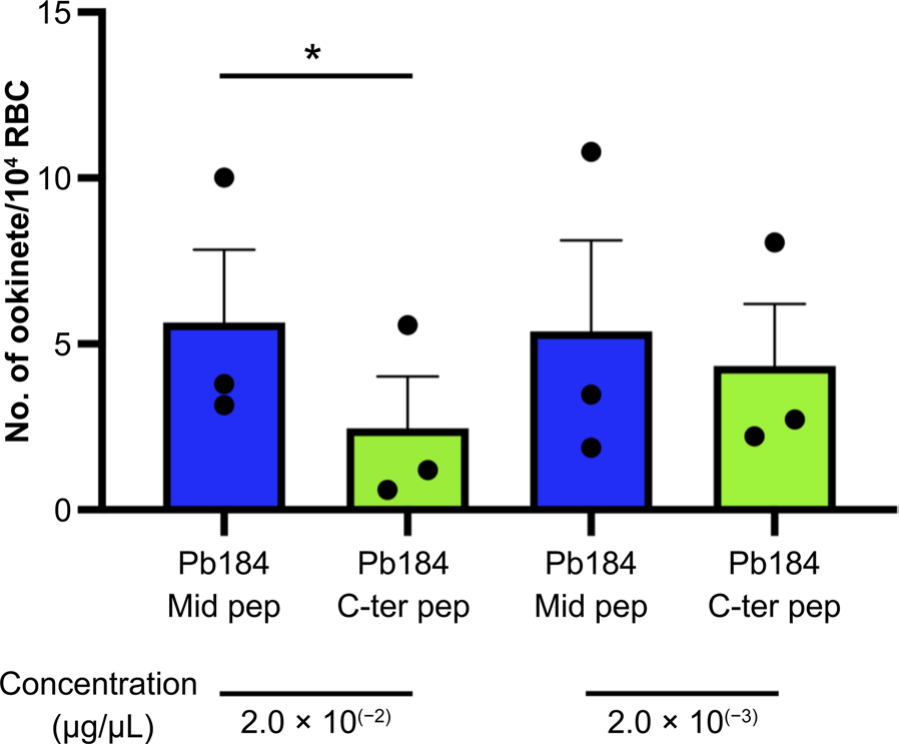
Ookinete differentiation assay. Pb184 C-ter-Pep, biotin-labeled Pb184 Mid-Pep or C-ter-Pep was added at the start of in vitro cultures, and ookinete numbers were counted 24 h later. Asterisk indicates a significant difference at *P < 0.05 (ratio paired-samples t-test). Pb184 C-ter Pep, Pb184 C-terminal peptide fragment; Pb184 Mid-pep, Pb184 middle peptide fragment; RBC, red blood cell

Finally, we examined whether biotin-labeled Pb184 C-ter-Pep could be involved in male–female gamete adhesion and observed that Pb184 C-ter-Pep reduced ookinete formation in a dose-dependent manner (Fig. 7; for a high concentration of peptides: ratio paired-samples t-test, *t*_(2)_ = 5.12, *P* = 0.036; for a low concentration of peptides: ratio paired-samples t-test, *t*_(2)_ = 1.01, *P* = 0.042). These results suggest that Pb184 C-ter-Pep inhibits male–female gamete adhesion.

## Discussion

In this study, we functionally analyzed the Pb184 protein. Our IFA revealed that Pb184 was expressed with a punctate localization on the surface of both male and female gametocytes and ookinetes. Our Pb184 C-terminal region (Pb184 C-ter)-targeting antibody inhibited zygote formation in vitro and reduced oocyst formation when fed to mosquitoes along with infectious blood. This result reveals that the Pb184 C-terminal region could be involved in male and female gamete adhesion during fertilization. We also proved that the biotin-labeled Pb184 C-ter-Pep binds to the female gametes and male gamete residual bodies. This peptide inhibited ookinete formation in vitro in a dose-dependent manner. These results suggest that Pb184, expressed on the male gamete cell membrane might bind to an unknown Pb184 receptor (Pb184 Rec) on the female gamete cell membrane, potentially contributing to gamete adhesion. Moreover, we performed fluorescence staining using the peptides on gamete samples that were not membrane permeabilized with Triton X-100. Interestingly, the acquired fluorescence was very faint, potentially because Pb184 Rec or its associated proteins, which might have been present on the cell membrane, were eluted during the permeabilization process prior to the α-tubulin staining upon peptide treatment. In addition, the binding of Pb184 C-ter-Pep to the residual body suggests the possibility that the interaction between male gametes and the residual body could be orchestrated by Pb184.

The gamete adhesion mechanism involves two major steps: gamete contact and membrane fusion. Proteins participating in gamete contact include P45/48, P230 and P47, all of which possess a 6-cys domain and function in both males and females. In addition, Pb22 and Pb115, which lack specific functional domains, are male-based for gamete contact [7–11]. In addition, PyMiGS, a protein expressed in the male gametocytes of the rodent malaria parasite *P. yoelii*, is involved in gamete adhesion [25, 26]. Membrane fusion is mediated by the male-specific HAP2/GCS1 protein [12–14]. Although Pb184 is presumably involved in the contact stage, its exact role in any of the stages remains unclear. A previous study investigated potential PfHAP2/GCS1 involvement in membrane fusion, by expressing PfHAP2/GCS1 in mammalian baby hamster kidney cells and monitoring eventual intercellular membrane fusions [14]. Similarly, it may be possible to express Pb184 and Pb184 Rec separately in cells to determine whether the occurrence of cell fusion indicates a role for Pb184 in membrane fusion.  

Clinical studies have reported simultaneous infections of multiple human malaria parasite species in a single individual, suggesting the coexistence of multiple parasite species in the mosquito midgut [27]. The first seven amino acids of the Pb184 C-terminal region are highly conserved, with certain eventual variations between species, suggesting a potential role for this region in species-specific reproductive isolation. Further research could deepen our understanding of membrane protein interactions between male and female gametes.

Two types of transmission-blocking vaccines (TBVs) have been developed: pre-TBVs target proteins expressed on the surfaces of male and female gametes and post-TBVs express proteins on zygotes and ookinetes [28, 29]. The anti-Pb184 C-ter Ab inhibited zygote differentiation in vitro and reduced oocyst formation, suggesting that Pb184 could be a potential candidate antigen for pre-TBV development. Moreover, the peptide used in this study might have potential applications as a peptide drug to inhibit malaria parasite differentiation. However, the experiment demonstrated that the antibody reduced the number of oocysts in mosquitoes by an average of only 20.3% on day 14 after blood-feeding and inhibited ookinete differentiation by a maximum of 50% in vitro, falling short of the 35% reduction required to achieve malaria elimination in certain models [30]. Therefore, for TBV applications, Pb184 might need to be combined with other transmission-blocking antigens. For peptide formulation-related applications, further C-terminal region optimization could be required to enhance its affinity with Pb184 Rec. For the latter purpose, this study focused on Pb184 receptor identification.

## Conclusions

In summary, we discovered that Pb184 functions in *Plasmodium* fertilization using the aforementioned C-terminal-recognized antibody and C-terminal-region peptide fragments. Further studies should aim at determining the role of Pb184 and addressing whether anti-Pb184 antibodies and peptide fragments could be transmission-blocking tools against malaria.

### Supplementary Information

Additional file 1: Primer list.

Additional file 2: The last 60 amino acids from the C-terminal region of the Pb184 sequence. Consensus are marked with “ * ” and “ : ” for identical and similar residues. Species infecting mice, humans, and primates are highlighted with white, gray, and black backgrounds, respectively.

Additional file 3: In vitro ookinete culture results with PBS, pre-immune antibody, anti-Pb184 mid antibody, and anti-Pb184 C-terminal antibody supplementation.

Additional file 4: Sequential fluorescent staining images from male gametocyte to gametes.The fluorescent images were captured at 0, 5, 10, and 15 min after exflagellation induction. Scale bar = 10 μm.

Additional file 5: Datasets.

## Data Availability

All data generated or analyzed in this study are included within the article and its Additional file 5.

## Abbreviations

aa: Amino acid
FBS: Fetal bovine serum
GFP: Green fluorescent protein
HRP: Horse rabbit protein
OSCP: Ookinete surface and oocyst capsule protein
PBS: Phosphate-buffered saline
TBV: Transmission-blocking vaccines
RT-PCR: Quantitative reverse transcription polymerase chain reaction
WT: Wild-type

## Declarations

### Permission to reproduce material from other sources

No third-party material requiring formal permission has been used in this study.
