# Supplementary material for: The C-terminal region of the Plasmodium berghei gamete surface 184-kDa protein Pb184 contributes to fertilization and male gamete binding to the residual body
Source: Parasit Vectors. 2024 Jul 13;17:304. doi: 10.1186/s13071-024-06374-7 (PMC11246575; doi:10.1186/s13071-024-06374-7)
Supplement: Supplementary file 1 — Additional file 1: Primer list. [file 13071_2024_6374_MOESM1_ESM.docx]

Additional file 1: Primer list

| Name | 5'→3' |
| --- | --- |
| Pb184 F.P | GCATGCCGAATTATATTCCTTCTT |
| Pb184 R.P | AGATACAATGTTCTGTTAATTGGCC |
| Pb18S rRNA F.P | AAGCATTAAATAAAGCGAATACATCCTTAC |
| Pb18S rRNA R.P | GGAGATTGGTTTTGACGTTTATGTG |
