# Supplementary figures and images for: The C-terminal region of the Plasmodium berghei gamete surface 184-kDa protein Pb184 contributes to fertilization and male gamete binding to the residual body
Source: Parasit Vectors. 2024 Jul 13;17:304. doi: 10.1186/s13071-024-06374-7 (PMC11246575; doi:10.1186/s13071-024-06374-7)

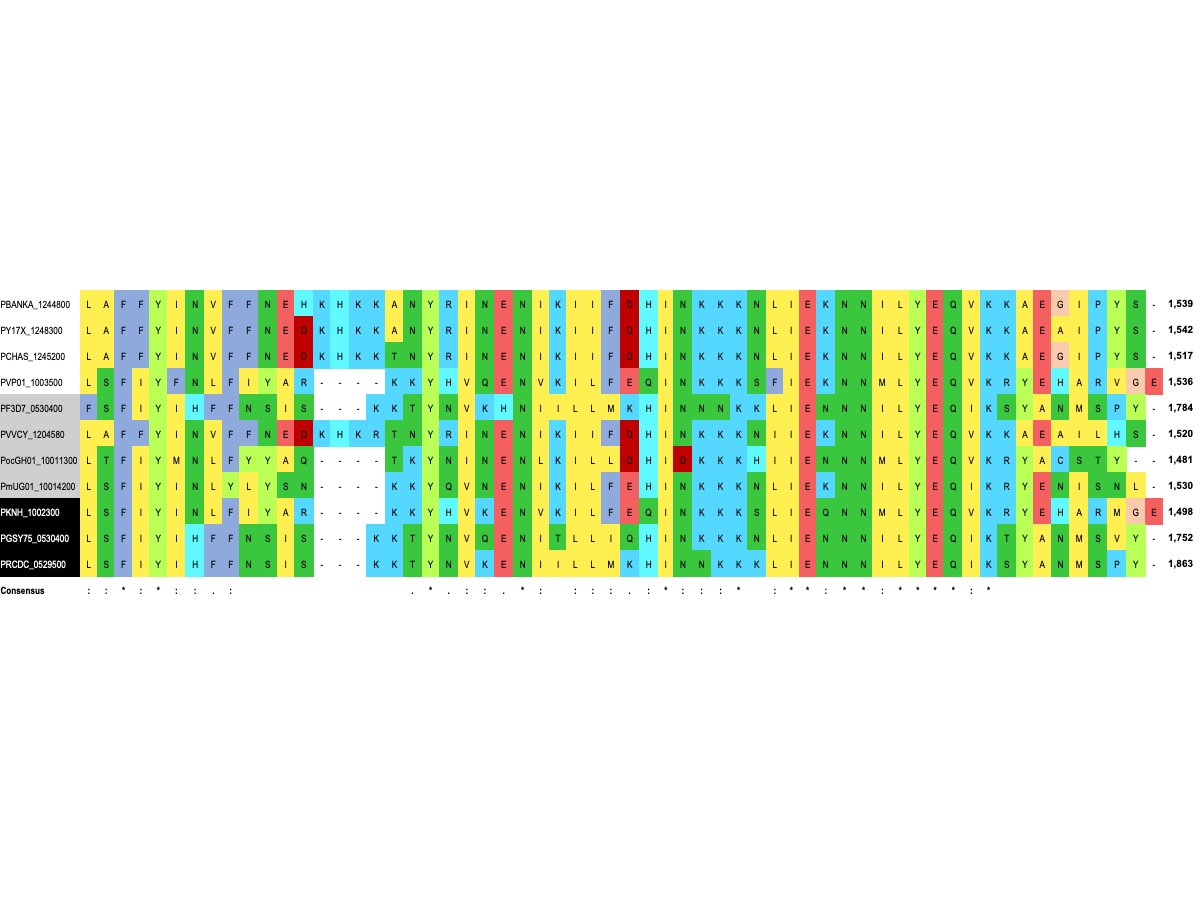

Supplement: Supplementary file 2 — Additional file 2: The last 60 amino acids from the C-terminal region of the Pb184 sequence. Consensus are marked with “ * ” and “ : ” for identical and similar residues. Species infecting mice, humans, and primates are highlighted with white, gray, and black backgrounds, respectively. [file 13071_2024_6374_MOESM2_ESM.tiff]

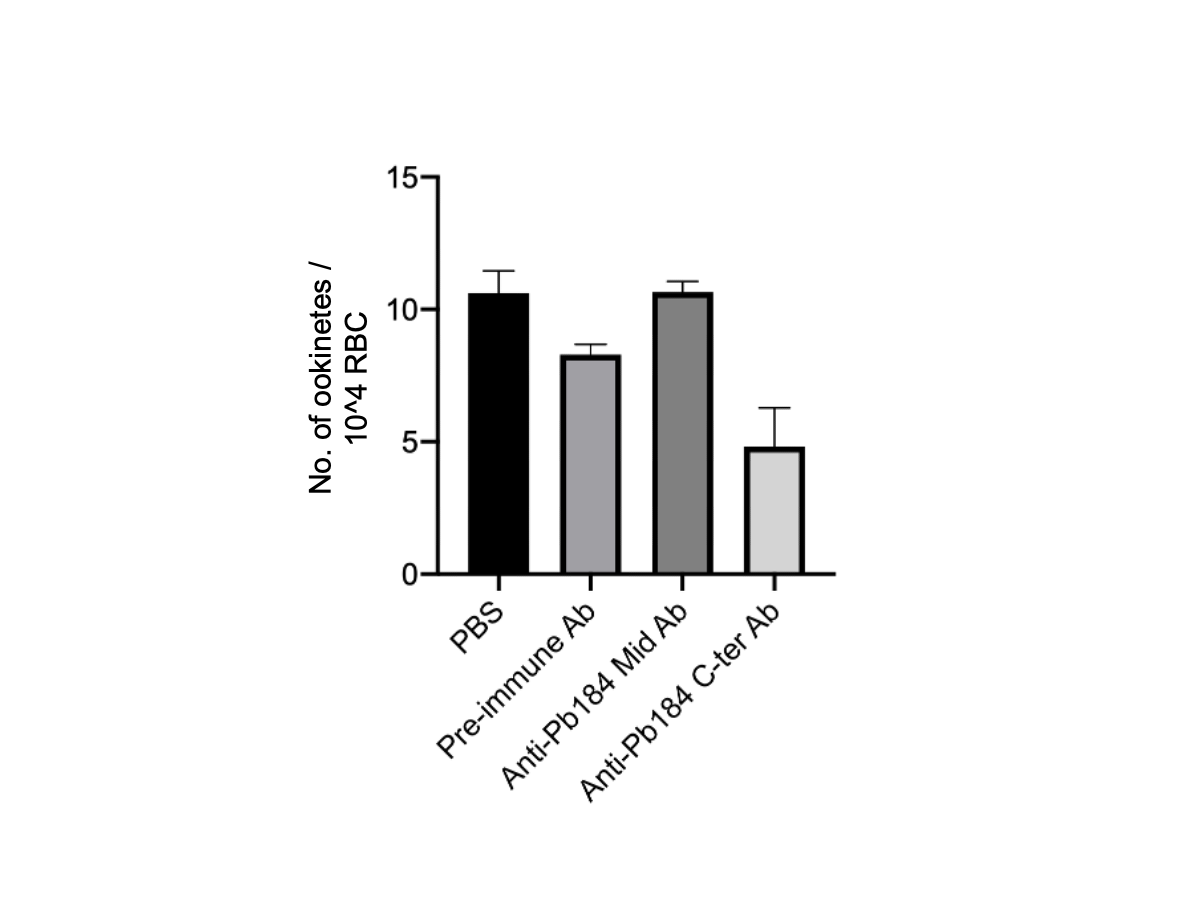

Supplement: Supplementary file 3 — Additional file 3: In vitro ookinete culture results with PBS, pre-immune antibody, anti-Pb184 mid antibody, and anti-Pb184 C-terminal antibody supplementation. [file 13071_2024_6374_MOESM3_ESM.tiff]

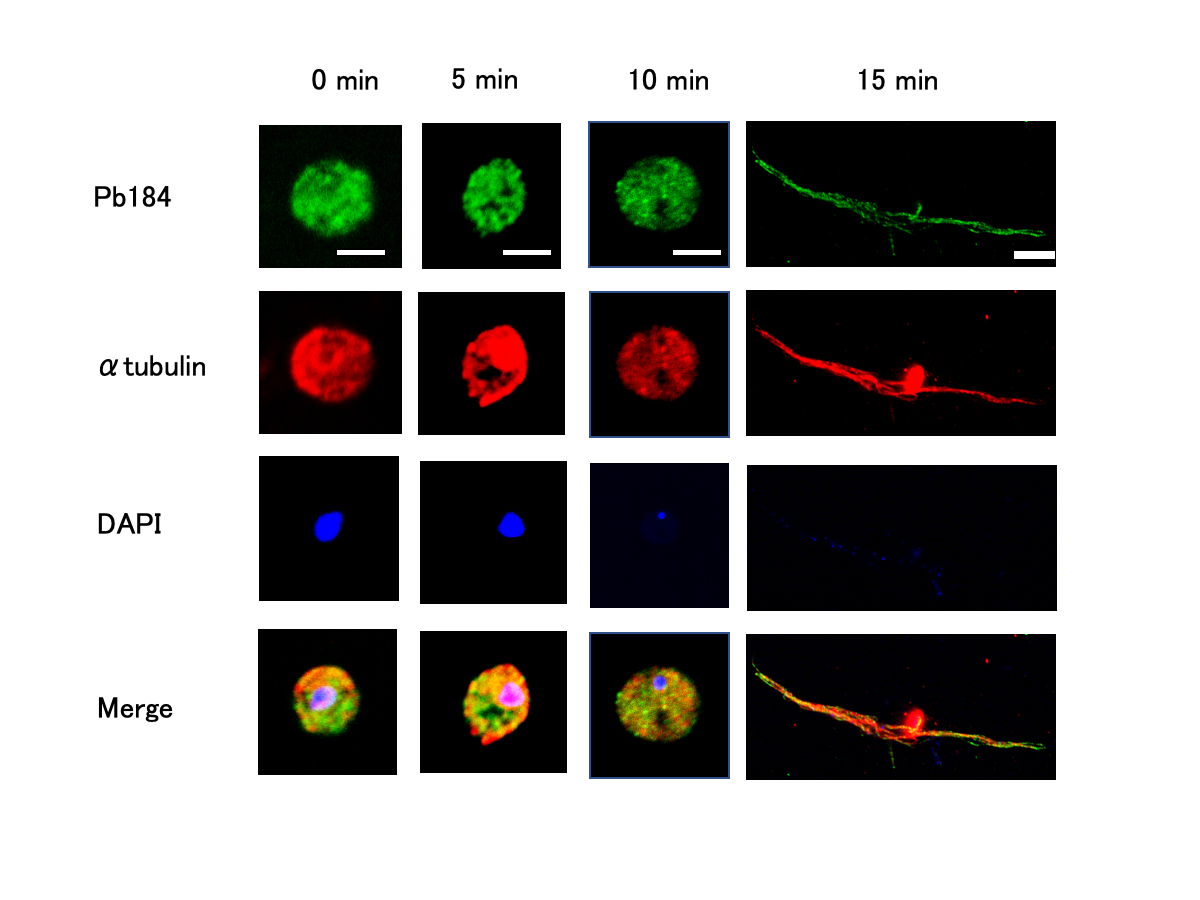

Supplement: Supplementary file 4 — Additional file 4: Sequential fluorescent staining images from male gametocyte to gametes.The fluorescent images were captured at 0, 5, 10, and 15 min after exflagellation induction. Scale bar = 10 μm. [file 13071_2024_6374_MOESM4_ESM.tiff]
